# Genome-Wide microRNA Expression Profiling in Human Spermatozoa and Its Relation to Sperm Quality

**DOI:** 10.3390/genes16010053

**Published:** 2025-01-04

**Authors:** Nino-Guy Cassuto, Florence Boitrelle, Hakima Mouik, Lionel Larue, Gwenola Keromnes, Nathalie Lédée, Laura Part-Ellenberg, Geraldine Dray, Léa Ruoso, Alexandre Rouen, John De Vos, Said Assou

**Affiliations:** 1 ART Unit, Drouot Laboratory, 75009 Paris, France; 2 Biology-Reproduction-Epigenetic-Environment-Development BREED, INRAE, Paris Saclay University, UVSQ, 78350 Jouy-en-Josas, France; 3 Faculty of Medicine and Pharmacy, University of Hassan II, Casablanca 28800, Morocco; 4 IVF ART Diaconesses Hospital, île de France, 75012 Paris, France; 5 IVF Center Bluets-Drouot, Les Bluets Hospital, 75012 Paris, France; 6 IVF ART Bluets Hospital, île de France, 75012 Paris, France; 7 AP-HP, Hôtel-Dieu, Sleep and Vigilance Center, Université Paris Cité, VIFASOM, ERC 7330, 75006 Paris, France; 8 Institute for Regenerative Medicine and Biotherapy (IRMB), University of Montpellier, INSERM, CHU Montpellier, 34295 Montpellier, France

**Keywords:** microRNA, male infertility, spermatozoa, biomarkers, high magnification

## Abstract

Background: Sperm samples are separated into bad and good quality samples in function of their phenotype, but this does not indicate their genetic quality. Methods: Here, we used GeneChip miRNA arrays to analyze microRNA expression in ten semen samples selected based on high-magnification morphology (score 6 vs. score 0) to identify miRNAs linked to sperm phenotype. Results: We found 86 upregulated and 21 downregulated miRNAs in good-quality sperm (score 6) compared with bad-quality sperm samples (score 0) (fold change > 2 and *p*-value < 0.05). *MiR-34* (FC × 30, *p* = 8.43 × 10^−8^), *miR-30* (FC × 12, *p* = 3.75 × 10^−6^), *miR-122* (FC × 8, *p* = 0.0031), *miR-20* (FC × 5.6, *p* = 0.0223), *miR-182* (FC × 4.83, *p* = 0.0008) and *miR-191* (FC × 4, *p* = 1.61 × 10^−6^) were among these upregulated miRNAs. In silico prediction algorithms predicted that miRNAs upregulated in good-quality sperm targeted 910 genes involved in key biological functions of spermatozoa, such as cell death and survival, cellular movement, molecular transport, response to stimuli, metabolism, and the regulation of oxidative stress. Genes deregulated in bad-quality sperm were involved in cell growth and proliferation. Conclusions: This study reveals that miRNA profiling may provide potential biomarkers of sperm quality.

## 1. Introduction

Infertility is a multifactorial pathology. Male infertility represents ~50% of all cases and is often due to abnormal sperm quality [1]. Today, the study of male infertility is largely limited to clinical examination, hormone assessment, karyotyping, and sperm functional parameter analysis [2]. However, the molecular analysis of spermatozoa could provide invaluable information on sperm quality to further understand male infertility. Comprehensive omics studies have recently been conducted in humans to better understand the complex processes related to spermatogenesis and male fertility at the gene methylation [3], transcript [4], and protein [5,6] levels. Post-transcriptional regulation of genes involved in spermatogenesis, particularly through miRNAs, is known to play a pivotal role in this process [7,8]. Nevertheless, despite substantial progress, the precise effects of miRNA dysregulation on sperm quality and male infertility remain to be elucidated.

We previously described a novel scoring system (ranging from 0 to 6) for living spermatozoa based on strict morphological criteria [9]. Spermatozoa with a score of 6 exhibit a normal head shape, characterized by a symmetrical nuclear membrane without invaginations or extrusions, the absence of vacuoles, and a normal base (the lower third of the sperm head extending to the neck where the centrosome is located). In contrast, spermatozoa with a score of 0 present head abnormalities, a defective base, and vacuoles. We found correlations between score, methylation pattern, and gene expression levels [3,10,11]. We hypothesized that such a score could also be influenced by the post-transcriptional regulations orchestrated by microRNAs (miRNAs). Indeed, sperm contain numerous small non-coding RNAs [12], including miRNAs [13,14].

MiRNAs are short, single-stranded non-coding RNA (typically ~22-nucleotide long) that post-transcriptionally repress the expression of their gene targets by binding to specific regions of target mRNAs, leading to decreased protein expression [15,16]. It is well established that miRNAs and their associated machinery play a critical role in regulating spermatozoa differentiation in the testes [17,18]. They are also involved in germ cell differentiation, a key aspect of spermatogenesis, which is a continuous process during postnatal life in males [19]. Therefore, it is important to investigate the effect of miRNA deregulation on male fertility. Moreover, some of these miRNAs could be candidate biomarkers of sperm quality.

Transcriptomic approaches, such as microarrays and next generation sequencing, have been used to identify the miRNA expression patterns in human testes and spermatozoa [20]. For instance, Barad et al. identified five tissue-specific miRNAs that are overexpressed in the testes [21]. Moreover, Yang et al., described the small RNA transcriptome of the testes in three healthy men [22]. Other groups used RNA sequencing (RNA-seq) to characterize the miRNA content in sperm samples [13,23]. Moreover, differential miRNA expression has been observed in men with normal versus impaired spermatogenesis [24] and in abnormal semen from infertile men versus the normal semen of healthy donors [25]. All these studies suggest that miRNA deregulation may affect spermatogenesis and contribute to infertility. The aim of this study was to investigate the miRNAome of spermatozoa classified based on their morphology (score 6 versus score 0) using miRNA microarrays and bioinformatics. The objective was to identify by in silico analysis, the putative gene targets and functional pathways affected by the miRNAs that are differentially expressed in good versus bad morphology spermatozoa.

## 2. Materials and Methods

### 2.1. Sample Collection and Preparation

The study received approval from the Institutional Review Board (IRB) of the French Language Andrology Society (IORG0010678) under the reference number 202304. The research was conducted at the Assisted Reproductive Technology (ART) Unit of the Drouot Laboratory in Paris, France. All participants provided written informed consent prior to their inclusion in the study. They were informed that their semen samples would be analyzed using high magnification and molecular biology techniques after the clinical tests. This procedure did not influence their treatment. No financial compensation was offered, and all data were anonymized. For this study, ten couples with infertility issues (female, male, or both) were enrolled at our ART unit (approved date April 2023).

Motile spermatozoa were separated and purified using a bilayer density gradient method. Conical tubes containing 90% and 45% Isolate Sperm Separation Medium (ref. 99264; Fujifilm Irvine Scientific, Santa Ana, CA, USA) were centrifuged at 300× *g* for 15 min. The supernatant was then carefully removed, and the resulting sperm pellets were rinsed with modified Human Tubal Fluid medium (ref. 90126; Fujifilm Irvine Scientific, Santa Ana, CA, USA). The pellets were subsequently centrifuged at 600× *g* for 10 min. After centrifugation, the pellets were resuspended in 500 μL of Tubal Fluid medium, and sperm cells were counted under a light microscope at high magnification. Only motile spermatozoa were selected based on strict morphological criteria and a validated scoring system, using a magnification of ×6100 [26]. Briefly, spermatozoa assigned “score 6” display a symmetrical nucleus, absence of vacuoles, and a normal base. In contrast, those with a score of 0 show head deformities, presence of vacuoles, and an abnormal base [9]. The analysis focused on 10 fresh sperm samples from 10 men (aged 36.7 ± 3.1 years), collected following a period of 2 to 3 days of sexual abstinence, as recommended by standard guidelines and categorized based on their sperm morphology scores [9]: five with a score of 6 and five with a score of 0. The patients are of Caucasian descent and reside in the Paris region (Île-de-France). They are in good health, live in a non-toxic environment, and are not exposed to endocrine disruptors. The characteristics of these samples are detailed in Supplementary Figure S1. Each sample was frozen and stored at −80 °C for subsequent miRNA isolation.

### 2.2. MiRNA Extraction

The RNA was extracted using a commercial kit (miRNeasy Kit, Qiagen, Hilden, Germany), according to the manufacturer’s instructions. Briefly, samples were thawed on ice, homogenized, and QIAzol^®^ Lysis Reagent (Qiagen, Hilden, Germany) was added to the sperm pellet and homogenized using a 20-G needle. The samples were then vortexed and incubated at room temperature for 5 min to ensure membrane dissociation. After incubation, 200 μL of chloroform was added. After vigorous shaking for 15 s and incubation at room temperature for 3 min, phases were separated by centrifugation at 12,000× *g* at 4 °C for 15 min. The upper aqueous phase (400 μL) was transferred to a gDNA eliminator column (Qiagen) to prevent DNA contamination of the purified RNA. Following centrifugation at 8000× *g*, at 4 °C for 30 s, the DNA-free phase was mixed with 600 μL of 100% ethanol by gentle pipetting. A total of 700 μL of the sample was transferred to an RNeasy MinElute spin column (Qiagen, Hilden, Germany) in a 2 mL collection tube and centrifuged at 8000× *g* at room temperature for 15 s to allow RNA binding to the membrane. This step was repeated with the remaining 300 μL of the sample using the same column. The column was then sequentially washed with 700 μL of RWT buffer, 500 μL of RPE buffer, and 500 μL of 80% ethanol, each time centrifuging at 8000× *g* at room temperature for the specified times and discarding the flow-through after each wash. The column was then dried by centrifuging at 15,000× *g* at room temperature for 6 min. RNA was eluted by adding 14 μL of nuclease-free water to the spin column membrane and centrifuging at 15,000× *g*, at room temperature, for 1 min. Total RNA (concentrations ranged from 26 to 127 ng/µL) was quantified using a NanoDrop^®^ One spectrophotometer (Thermo Fisher Scientific, Wilmington, DE, USA), and its integrity was assessed using an Agilent 2100 Bioanalyzer (Agilent Technologies, Palo Alto, CA, USA). All samples were stored at −80 °C for further analysis.

### 2.3. MiRNA Microarray Analysis

Microarray technology is a very powerful high-throughput tool for monitoring the expression of thousands of miRNAs at once in samples processed in parallel in a single experiment [27]. The profiling of miRNA expression was carried out using the GeneChip miRNA 4.0 arrays from Affymetrix^®^ (Thermo Fisher Scientific, Courtaboeuf, France). To produce biotin-labeled RNA, the Affymetrix^®^ FlashTag™ Biotin HSR RNA Labeling Kit (Thermo Fisher Scientific, Courtaboeuf, France) was employed. In summary, 200 ng of extracted RNA was subjected to poly-A tailing at the 3′-end, followed by the attachment of a biotin-labeled 3DNA molecule facilitated by a DNA ligase. The labeled RNA samples were then hybridized onto the GeneChip miRNA 4.0 arrays in an Affymetrix^®^ Oven 455 (Thermo Fisher Scientific, Courtaboeuf, France) under controlled conditions (48 °C, 60 rpm rotation) for 18 h. Following hybridization, the arrays were washed and stained using the GeneChip™ Hybridization, Wash, and Stain Kit (Thermo Fisher Scientific, Courtaboeuf, France) on an Affymetrix^®^ Fluidic Station 450. The arrays were finally scanned with the Affymetrix^®^ GeneChip Scanner 3000 7G. Each sample was independently analyzed on a dedicated GeneChip miRNA array.

### 2.4. Data Analysis and Bioinformatics Processing

The raw .CEL files were produced using the Affymetrix^®^ GeneChip Command Console Software (version 4.0, Thermo Fisher Scientific) and subsequently processed in the Affymetrix^®^ Transcriptome Analysis Console (TAC) software (version 4.0, Thermo Fisher Scientific). Intensity normalization was performed using the robust multi-array average (RMA) method. Comparative analysis was conducted between S6 and S0 samples, with p-values adjusted according to the Benjamini–Hochberg FDR correction. miRNAs showing an FDR < 0.05 and a fold change (FC) of ≥2.0 or ≤−2.0 were considered significantly upregulated or downregulated. Principal component analysis (PCA) and hierarchical clustering were applied to evaluate sample grouping based on the differentially expressed miRNAs, using the Transcriptome Analysis Console software v4.0. Previously published DNA sequencing data [3] were utilized to investigate the methylome of S6 and S0 samples, focusing on identifying differential methylation at miRNA target genes. The raw sequencing data were aligned to the human reference genome (Homo sapiens, GRCh37/hg19) using BSMAP software (v2.89). Uniquely mapped reads without PCR duplicates were processed for bigwig file generation using the deeptools bamCoverage tool (v3.3.5) [28] with the following parameters: --binSize 1 --normalizeUsing RPGC --effectiveGenomeSize 2701495761 --scaleFactor 1.0 --minMappingQuality ‘1’ --ignoreForNormalization chrX chrM.

### 2.5. Integrative Analysis of miRNA and mRNA Expression

IPA (Ingenuity Pathway Analysis, www.ingenuity.com (accessed on 2 June 2024)) was used to identify interactions between miRNAs and their mRNA targets. miRNAs (and their expression values) and target genes were uploaded into the software where each gene symbol was mapped to its corresponding gene object in the Ingenuity Pathways Knowledge Base. The sources for validated miRNA data included TarBase, miRecords, and TargetScan, along with miRNA–mRNA interactions from peer-reviewed biomedical literature. Only interactions between miRNAs and mRNAs validated by experimental observations were selected. Gene networks were algorithmically constructed based on connectivity and given a score to rank networks according to their relevance to the genes in the input dataset; however, this score does not necessarily indicate network quality or significance. The score reflects the number of focus genes in the network and network size, estimating the relevance to the initial list of focus genes. The S6-miRNA target genes were imported into IPA, where each gene identifier was mapped to a global molecular network derived from data in the Ingenuity Pathways Knowledge Base. Networks of these genes were generated based on connectivity, and a network score was calculated using a hyper-geometric distribution with a right-tailed Fisher’s exact test, considering a *p*-value of <0.05 as significant. Functional enrichment and pathway analyses were carried out using ShinyGO [29] and GSEA (http://www.broadinstitute.org/gsea/ (accessed on 7 June 2024)). Gene Ontology (GO) terms were classified into three main categories: biological processes, molecular functions, and cellular components. Fisher’s exact test was applied to evaluate the enrichment of genes in specific terms, with a significance threshold set at an adjusted p-value of <0.05. Additionally, pathway analysis of target mRNAs was performed to highlight key pathways, utilizing the IPA software (version 24.0) and the KEGG database. Using GTEx RNA-seq data integrated within IPA, we examined gene expression across thirty tissues and identified genes that are significantly downregulated in the testis.

### 2.6. Statistical Analysis

Only genes (miRNAs) with an adjusted *p*-value < 0.05 and a fold change >2 were selected as differentially expressed and included in the analysis. Fisher’s exact test and unadjusted *p* values were used for the IPA.

## 3. Results

### 3.1. MiRNAs Expression Patterns in Score 6 and Score 0 Spermatozoa

**Figure 1:**
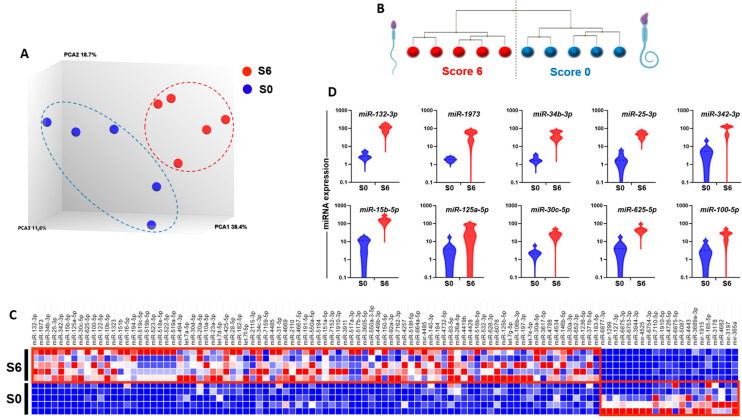
Differences in the global miRNA expression profiles of S6 and S0 sperm samples. (**A**). Unsupervised 3D PCA representing the miRNA expression patterns of S6 spermatozoa (*n* = 5 samples) and S0 spermatozoa (*n* = 5 samples). Each sample was analyzed using the GeneChip^®^ miRNA 4.0 Array. Red dots, S6 samples; blue dots, S0 samples. (**B**). Hierarchical clustering of the samples using the differentially expressed miRNAs with the highest variation. S6 and S0 samples (n = 5/each group) are clustered in two distinct groups. (**C**). Heat map of the S6 and S0 miRNA signatures based on the 107 miRNAs that are differentially expressed between S6 and S0 samples. Each column corresponds to a specific miRNA, and each row represents a sperm sample. The color scale reflects the relative miRNA expression levels, with red indicating higher expression and blue indicating lower expression. (**D**). Violin plots showing the expression of the top 10 upregulated miRNAs in S6 samples based on the TAC analysis of the microarray data. S6: good quality samples, S0: bad quality samples.

The GeneChip™ miRNA 4.0 Array (Thermo Fisher Scientific, Courtaboeuf, France) was used to examine the miRNA expression profiles in ten sperm samples (five with score 6, S6, and five with score 0, S0). The collected data were then analyzed by PCA and hierarchical clustering to visually summarize the normalized miRNA expression profiles and detect similarities and differences among samples (Figure 1A,B). These results showed a distinct directionality of S6 and S0 samples, suggesting that their miRNA profiles were significantly different. Indeed, a comparison of their miRNA profiles with the Transcriptome Analysis Console (TAC) software (version 4.0, Thermo Fisher Scientific) (with a 2-fold change threshold and a false discovery rate, FDR, <5%) identified 107 differentially expressed miRNAs between S6 and S0 samples. Specifically, 21 miRNAs were downregulated, and 86 miRNAs were upregulated in the S6 samples. The list of differentially expressed miRNAs (up/downregulated) between S6 and S0 samples are in Supplementary Tables S1 and S2. The heatmap based on these differentially expressed miRNAs separated perfectly the “S6-miRNA signature” and “S0-miRNA signature” (Figure 1C). In the “S6-miRNA signature”, miRNAs with high fold change (FC) included *miR-132-3p* (FC × 41.5), *miR-1973* (FC × 32.4), *miR-34b-3p* (FC × 30.2), *miR-25-3p* (FC × 27.3), *miR-342-3p* (FC × 24.8), *miR-15b-5p* (FC × 14), *miR-125a-5p* (FC × 13.8), *miR-30c-5p* (FC × 12.1), *miR-625-5p* (FC × 9.7), *miR-100-5p* (FC × 8.3), *miR-122-5p* (FC × 8), and *hsa-miR-10b-5p* (FC × 8). Conversely, the “S0-miRNA signature” included few miRNAs with high FC, particularly *miR-6877-3p* (FC × 17.4), *miR-1299* (FC × 10.2), *miR-127-3p* (FC × 7.8), *miR-6875-3p* (FC × 7.7), *miR-6753-3p* (FC × 5.7), *miR-3944-3p* (FC × 5.7), and *mir-4525* (FC × 4.7). The expression levels of the miRNAs that best represented the “S6-miRNA signature” are shown in Figure 1D.

### 3.2. Regulatory Roles of the miRNAs in the S6-miRNA Signature

**Figure 2:**
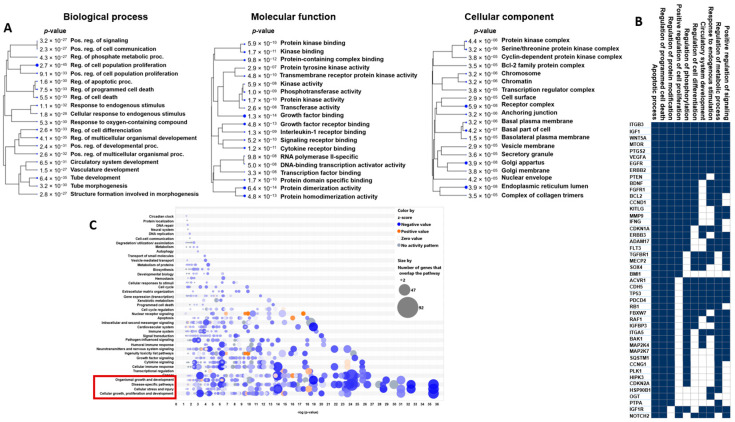
Analysis of GO terms associated with S6-miRNA targets and their functions. (**A**). Analysis of significantly represented GO terms. Pathway enrichment analyses were carried out using the human gene names of S6-miRNA targets. The size of the blue dots reflects the degree of enrichment, with larger dots representing more significant *p*-values. (**B**). GSEA was conducted using the S6-miRNA targets. The heat map illustrates the clustering of genes within the leading-edge subsets, emphasizing the dynamic expression of genes associated with programmed cell death regulation, phosphorylation, positive regulation of cell proliferation, and metabolic processes. Genes are shown on the vertical bars colored from deep blue (top rank) to blank (lowest rank). (**C**). Bubble plot of the overlapping canonical pathways associated with S6-miRNA targets. The circle size reflects the number of genes involved in the pathway. The canonical pathways were categorized into various types based on the IPA database.

Then, the Ingenuity Pathway Analysis (IPA) identified 910 mRNAs targeted by the 86 miRNAs upregulated in S6 samples and 15 mRNAs targeted by the 21 miRNAs downregulated in S6 samples (Supplementary Tables S3 and S4). Given the low number of targets by miRNAs of the S0-miRNA signature, the next analyses focused only on the upregulated S6 miRNAs. Analysis of the Gene Ontology (GO) terms associated with the “S6-miRNA signature” revealed that the miRNA targets were particularly implicated in specific biological processes, e.g., regulation of cell proliferation (GO:0042127), cellular response to chemical stimulus (GO:0070887) and regulation of programmed cell death (GO:0043067), molecular functions, e.g., protein kinase activity (GO:0004672), phosphotransferase activity (GO:0016773), growth factor binding (GO:0019838)], and cellular components (e.g., membrane, nucleus) (Figure 2A). Next, Gene Set Enrichment Analysis (GSEA) and IPA were used to identify upstream transcriptional regulators, significant canonical pathways, and relevant gene networks. GSEA showed the enrichment of gene sets associated with regulation of programmed cell death, regulation of phosphorylation, positive regulation of cell proliferation, and regulation of metabolic process (Figure 2B). IPA indicated that the top canonical pathways were linked to cellular growth, proliferation and development, cellular stress, and organismal growth (Figure 2C).

**Figure 3:**
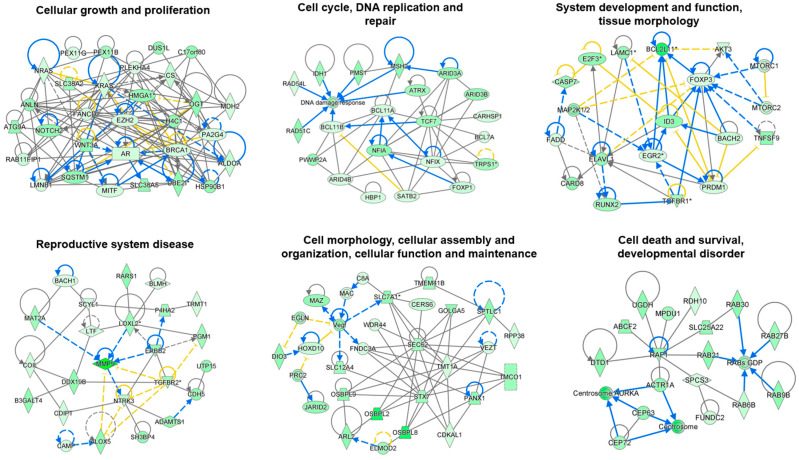
Top-ranked functional networks of the S6-miRNA target genes. Top networks identified by IPA of S6-miRNA target genes related cell growth and proliferation, cell cycle regulation, DNA replication and repair, system development and function, tissue morphology, reproductive system disorders, cell morphology, cellular assembly and organization, cellular function and maintenance, cell death and survival, and developmental disorders. Green nodes represent genes regulated by S6-miRNAs. Dashed lines represent indirect relationships, while solid lines indicate direct molecular interactions. Within each network, the edge types are defined as follows: a line without an arrowhead signifies binding only, a line ending with a vertical bar represents inhibition, and a line with an arrowhead indicates an “acts on” relationship. *: indicate that several gene identifiers in the dataset file correspond to a single gene in the Global Molecular Network.

IPA was also used to examine the interactions within the 910 mRNAs targeted by S6-miRNAs. This analysis identified six networks associated with (i) cellular growth and proliferation, (ii) cell cycle, DNA replication and repair, (iii) system development and function, tissue morphology, (iv) reproductive system disease, (v) cell morphology, cellular assembly and organization, cellular function and maintenance, and (vi) cell death and survival and developmental disorder (Figure 3).

**Figure 4:**
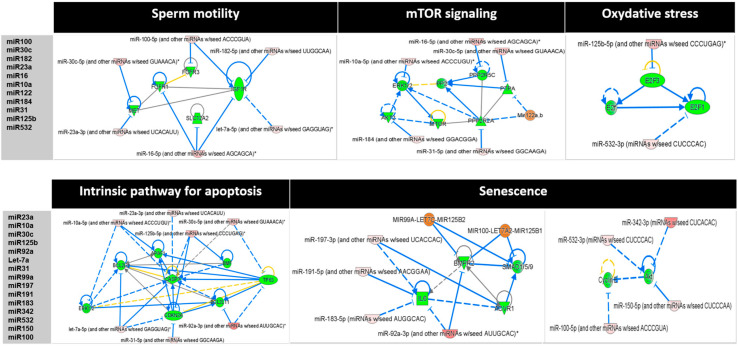
Networks of the S6-miRNA target genes. The IPA tool was used to generate the networks based on the predicted miRNA–mRNA interactions. Pink nodes represent the miRNAs upregulated in S6 samples and green nodes represent the genes targeted by S6-miRNAs. Solid lines represent direct interactions and dashed lines indirect interactions. *: indicate that several gene identifiers in the dataset file correspond to a single gene in the Global Molecular Network.

Then, the IPA miRNA Target Filter was used alongside genes predicted to be targets of S6-miRNAs to identify potential miRNA–mRNA interactions. This analysis predicted that S6-miRNAs interacted with protein-coding genes involved in intercellular communication in sperm motility, mammalian target of rapamycin (mTOR) signaling, oxidative stress, intrinsic pathway for apoptosis, and senescence (Figure 4). For instance, *miR-100*, *miR-30c*, *miR-182*, *miR-23a*, and *miR-16* interacted with genes that participate in the regulation of sperm motility, such as *FGFR1*, *FGFR3*, *MET*, *IGF1R*, and *SLC12A2*. Moreover, *miR-30c*, *miR-16*, *miR-10a*, *miR-122a*, *miR-184*, and *miR-31* functionally interacted with *mTOR*, *AKT2*, *ERK1/2*, *PP2*, *PPP2R5C*, *PPP2R2A*, and *PTPA*. Two miRNAs (*miR-125b* and *miR-532*) formed a tightly connected network with *E2F3* and *E2F1* and were central in the regulation of oxidative stress. In addition, *miR23a*, *miR-10a*, *miR-30c*, *miR-125b*, *miR92a*, *Let-7a*, *miR-31*, *miR-99a*, *miR-197*, *miR191*, *miR-183*, *miR-342*, *miR-532*, *miR-150*, and *miR-100* interacted with genes implicated in the regulation of programmed cell death such as *BCL2L11*, *BCL2L1*, *CASP3*, *CDKN2A*, *ERK1/2*, *BMPR2*, *SMAD1/5/9*, *Cyclin E*, and *AKT2*. Altogether, these results are in line with the major processes that occur during spermatogenesis and that influence sperm quality.

### 3.3. Methylation of Key Genes Targeted by S6-miRNAs in Not Different in S6 and S0 Sperm Samples

**Figure 5:**
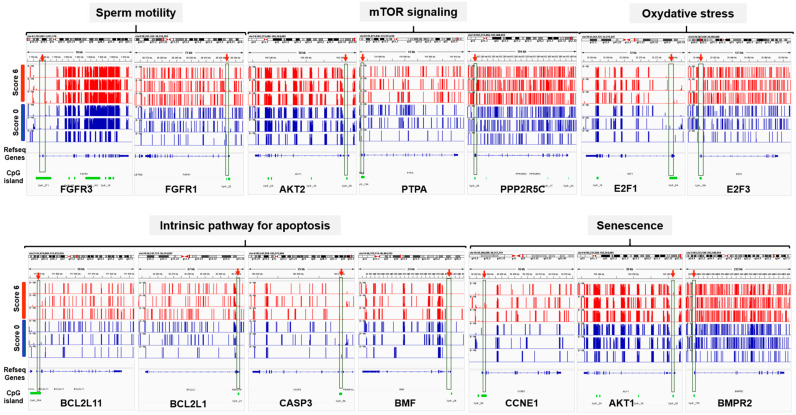
The promoters of the predicted S6-miRNA target genes are not differentially methylated. Integrative Genome Viewer snapshots illustrating the methylation levels at individual CpG sites (0–100%) across the examined genes. Each promoter region (red arrow) overlaps with a CpG island (green box).

To explore the biological connections between the predicted S6-miRNA target genes and their corresponding DNA methylation profiles, S6 and S0 semen samples were analyzed by whole-genome bisulfite sequencing. This technique allows precisely mapping the methylation patterns genome wide. The present analysis focused particularly on CpG islands (i.e., cytosine-guanine rich regions often associated with gene promoters and playing a crucial role in gene regulation) located within the promoters of the predicted S6-miRNA target genes. Many of these genes are implicated in key pathways, such as sperm motility (*FGFR3*, *FGFR1*), mTOR signaling (*AKT2*, *PTPA*, *PPP2R5C*), oxidative stress (*E2F1*, *E2F3*), intrinsic pathway for apoptosis (*BCL2L11*, *BCL2L1*, *CASP3*, *BMF*). and senescence (*CCNE1*, *AKT1*, *BMPR2*). The promoters of these genes were consistently hypomethylated in both S6 and S0 samples (Figure 5).

Hypomethylation of promoter regions is typically associated with active gene expression because it allows the binding of transcription factors and the initiation of transcription. The consistent hypomethylation observed in all samples suggests that the mRNA substrate is available in both S6 and S0 samples, but that these pathways are post-transcriptionally inhibited by miRNAs upregulated in S6 samples. This insight enhances our understanding of the complex regulatory networks at play in spermatogenesis, highlighting the potential role of miRNAs as key regulators of gene expression, leading to the impaired phenotype observed in S0 samples.

### 3.4. Targets of miRNAs Upregulated in S6 Samples Are Downregulated in Testes

**Figure 6:**
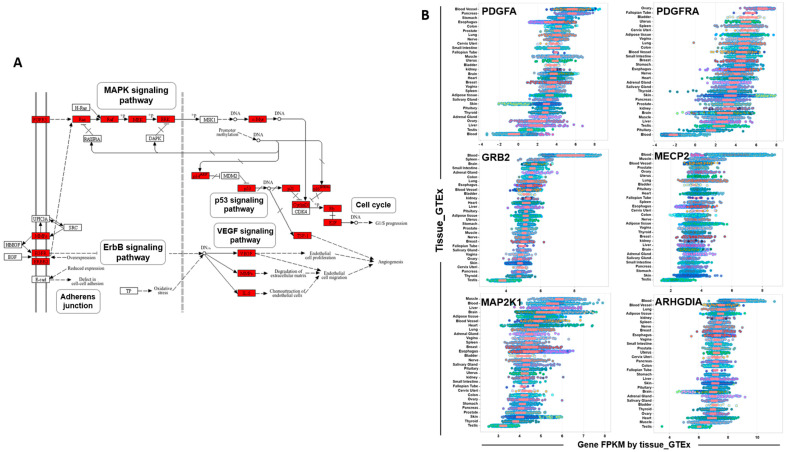
Enrichment of S6-miRNA targets in critical signaling pathways and their expression in testes. (**A**). Pathway analysis (KEGG pathway) using the Pathview server (https://pathview.uncc.edu/ (accessed on 17 June 2024)). Highlighted genes are pathway components identified as targets of S6 miRNAs. (**B**). Expression profile of candidate genes in various human tissues. Expression levels (in Log2 RPKM) of *PDGFA*, *PDGFRA*, *GRB2*, *MECP2*, *MAP2K1*, *ARHGDIA*, and *MET* in 30 tissues from GTEx. For each gene, the colored circle corresponding to each tissue represents the RPKM value averaged across all samples within that tissue. RPKM stands for reads per kilobase of transcript per million mapped reads.

To establish a connection between the identified S6-miRNAs and their associated pathways, a comprehensive bioinformatics analysis was conducted using Kyoto Encyclopedia of Genes and Genomes (KEGG) and IPA. This analysis showed that the genes targeted by miRNAs upregulated in S6 samples were significantly enriched in the PDGF, EGF, and MAPK signaling pathways (Figure 6A). Then, RNA-seq data from the Genotype-Tissue Expression (GTEx) repository [30,31] were used to analyze the expression levels of genes associated with these signaling pathways in thirty normal tissues, including testis. This analysis revealed that several key genes, namely *PDGFA*, *PDGFRA*, *GRB2*, *MECP2*, *MAP2K1*, and *ARHGDIA*, were significantly downregulated in testis tissue compared with other tissues (Figure 6B). This suggests that these genes may play a crucial role in the miRNA-mediated regulation of signaling pathways. Specifically, these pathways include the mTOR, p53, and MAPK signaling pathways, all of which may be regulated in a context-specific manner in the testis and are targeted by S6-miRNAs.

## 4. Discussion

The microarray technology allowed us to precisely quantify and characterize the relative abundance of a wide range of miRNAs in spermatozoa classified in function of their morphology, which is crucial for intracytoplasmic sperm injection (ICSI) [9,10]. The microarray platform provides greater robustness and stability as it is normalized to the total transcriptome expression measured in the array. This study offers a comprehensive analysis of the miRNA content in S6 and S0 sperm samples and uncovered the relationship between miRNA expression and sperm morphology. Analysis of the microarray data indicated differential miRNA expression levels in the two sample types. Notably, the number of upregulated miRNAs was higher in S6 than S0 samples. The “S6- miRNA signature” included *miR-132*, *miR-191*, *miR-34b*, *miR-34c*, *miR-449*, *miR-122*, *miR-146b*, *miR-93*, *let-7a*, *miR-15b*, *miR-16*, *miR-182*, *miR23a*, *miR-10b*, *miR-25*, *miR-26a*, *miR-30c*, *miR-197*, *miR-342*, *miR-125a*, *miR-92a*, and *miR-100*. As we discuss below, these miRNAs are involved in various aspects of sperm quality, including motility, morphology, energy metabolism, apoptosis, spermatogenesis steps, and fertility potential. Abnormal expression of these miRNAs can lead to suboptimal sperm function, resulting in male infertility or reduced reproductive success. Understanding their specific roles and mechanisms could provide valuable insights into male fertility for the development of potential therapeutic interventions for infertility. For instance, miR-132 is regulated by hormones in testis steroidogenic cells and plays a role in controlling steroidogenesis in Y1 adrenal cells by targeting StAR [32]. Wang et al. [33] suggested that tissue-specific regulation of *miR-132* is involved in metabolic disorders and sperm function in male mice. Hua et al. [34] identified *miR-132-3p* and *miR-191-5p* as potential markers of sperm quality in in vitro fertilization (IVF). Another study reported that fertilization, effective embryo, and high-quality embryo rates are higher when using sperm with high *miR-191-5p* expression, suggesting that this miRNA could serve as a biomarker for identifying high-quality sperm for IVF [35].

A recent study compared 106 semen samples (40 with normozoospermia, 47 with asthenozoospermia, and 19 with oligozoospermia) [36] and found that *miR-34b-5p*, *miR-34c-3p*, and *miR-34b-3p* were significantly less expressed in semen samples of patients with oligozoospermia than in those with normozoospermia [36]. Similarly, dysregulation of *miR-449* has been associated with male infertility due to impaired sperm motility [37]. Moreover, *miR-122-5p* is abundantly and consistently expressed in the testes and sperm of fertile males, indicating its potential as a biomarker [20]. Abnormalities in the regulation of this miRNA in the testes and sperm have been linked to germ cell arrest and defects in sperm formation, highlighting its important role in spermatogenesis [20]. Molecular studies have shown that *miR-122-5p* plays a crucial role in spermatogenesis by regulating the expression of *TNP2* (transition protein 2), which is essential for proper chromatin compaction during spermiogenesis [38]. Aberrant chromatin condensation in spermatozoa with vacuoles, linked to improper histone-to-protamine replacement involving TNP2, reflects structural anomalies associated with less compacted chromatin [39]. These defects correlate with sperm quality scores, where a score of 0 indicates severe abnormalities and 6 reflects optimal chromatin compaction. Abu-Halima et al. [40] demonstrated that *miR-34b* and *miR-122* are differentially expressed between subfertile individuals and controls, highlighting their potential as biomarkers. Combining these miRNAs with conventional tests has been shown to enhance diagnostic accuracy, particularly in distinguishing cases of non-obstructive azoospermia. Furthermore, sperm-borne *miR-34c-5p* is thought to initiate the first cleavage divisions in mice [41] and has been positively linked to ICSI outcomes, including the generation of high-quality embryos, successful implantation, pregnancy, and live birth in humans [42].

In humans, *miR-34c-5p* expression is an indicator of sperm viability and quality [40]. Based on these findings, *miR-34c-5p* can be considered a candidate non-invasive biomarker of sperm and embryos quality, although extensive validation in a large cohort is necessary. Wang et al. [43] reported that *miR-34c-5p*, *miR-122*, and *miR-146b-5p* are markedly decreased in the seminal plasma of men with asthenozoospermia compared with men with normozoospermia. Salas-Huetos et al. [44] demonstrated that the downregulation of *miR-93-3p* and *miR-34b-3p* is strongly linked to unexplained male infertility. In a follow-up study, they analyzed normozoospermic sperm from infertile and fertile men, identifying 57 miRNAs with differential expression between the two groups [45] among which *miR-93-3p*, *let-7a-5p*, *miR-15b-5p*, and *miR-191-5p* were also detected in S6 samples. Gholami et al. [46] identified *miR-182-5p* as a component of a regulatory network associated cysteine-rich secretory protein 3 (CRISP3) isoforms in the sperm of men with teratozoospermia. They suggested that *miR-182-5p* upregulation could serve as a potential biomarker of teratozoospermia. These studies and the present work propose a correlation between sperm phenotype and miRNA expression profile. Among the miRNAs not included in the S6 signature, *let-7a-5p* stands out as particularly worthy of investigation for its role in spermatogenesis, given its correlation with post-fertilization development and embryo quality [47]. Jung et al. [48] found that *let-7a* expression levels are consistently elevated in germ cells, suggesting a potential role in inhibiting cell proliferation.

Another study in 192 patients with idiopathic male infertility indicated that *let-7a* expression in the seminal plasma is reproducible and stable. Aberrant *let-7a* upregulation may be an indicator of spermatogenesis failure [49]. However, the specific function of let-7a in sperm remains unclear [50]. *Let-7a* and *miR-23a* are predicted to target caspase-3 and B-cell chronic lymphocytic leukemia/lymphoma-2 (BCL-2), both of which are involved in apoptosis. BCL-2 plays a vital role in the development of male germ cells by regulating spermatogonial apoptosis. An imbalance of this gene in transgenic animals disrupts spermatogenesis, leading to male subfertility and infertility [51,52]. *MiR-15* and *MiR-16* are pro-apoptotic miRNAs that target *BCL-2*, thereby promoting apoptosis in chronic lymphocytic leukemia [53]. However, their roles and underlying mechanisms in spermatogenesis are unknown. Studies on sperm apoptosis reveal that apoptotic markers in sperm closely resemble those in somatic cells, including caspase activation and DNA damage [54].

Mitochondria, key players in the intrinsic apoptotic pathway, are crucial in triggering apoptosis in sperm. Apoptosis induction in human sperm increases caspase 3 activity and reduces sperm motility, which is linked to lower sperm quality, higher DNA fragmentation, and decreased fertilization potential [55,56]. These findings support the role of the mitochondrial intrinsic pathway in regulating sperm apoptosis. Senescence also may increase apoptosis. Our findings suggest that some genes involved in senescence might be regulated by S6-miRNAs. The impact of senescence is often overlooked in studies on male infertility. Reduced levels of *miR-10b* were also found in men with asthenozoospermia, and *miR-34c* and *miR-122* were significantly downregulated in men with oligo-asthenozoospermia [57,58]. *MiR-15b* is predicted to target the mRNA of isocitrate dehydrogenase 3 (*NAD+*) alpha (*IDH3A*). Reduced expression of *IDH3A* impairs sperm motility by affecting sperm energy metabolism [59]. *MiR-26a* targets EH domain-containing 1, a protein crucial for pre- and post-natal development and spermatogenesis [60]. *MiR-30c* and *miR-197* are predicted to target the mRNA of activin receptor type-1 (*ACVR1*), a protein that regulates various aspects of cell development essential for reproductive fitness. Notably, *Acvr1* is significantly upregulated during mouse testis proliferation [61]. These findings suggest that S6-miRNAs play a significant role in testis development, spermatogenesis and/or post-fertilization development. Li et al. [62] demonstrated that *miR-125b* knockout in the testis disrupts PAP gene expression, reduces mitochondrial copy numbers, and impairs sperm quality, highlighting its essential role in maintaining sperm health through mitochondrial regulation. Similarly, Liu et al. [63] showed that *miR-100* regulates Sertoli cell proliferation, DNA synthesis, and apoptosis by binding to *SGK3*, emphasizing its importance in spermatogenesis. Furthermore, Gupta et al. [64] showed that the FSH-induced decrease in *miR-92a* expression in Sertoli cells is essential for germ cell differentiation and fertility, highlighting the critical role of *miR-92a* in regulating germ cell development. These studies suggest that miRNAs interact in complex networks to regulate spermatogenesis, with individual miRNAs potentially acting independently or compensating for one another. The abundance of these miRNAs in sperm, especially concerning sperm morphology, suggests their potential as biomarkers for sperm quality. However, conflicting findings exist regarding their roles, with some studies indicating they are fingerprints of spermatogenesis, while others suggest they are mainly transferred during epididymal maturation. Further research is needed to clarify these mechanisms and better understand miRNA regulation in male fertility.

The analysis of DNA methylation profiles in S6 and S0 sperm samples provides important insights into the regulation of genes critical for spermatogenesis. Promoter regions of genes involved in key pathways, such as sperm motility, mTOR signaling, oxidative stress, apoptosis, and senescence, were consistently hypomethylated in both sample groups. This hypomethylation, typically associated with active transcription, suggests that the mRNA substrates for these genes are present in both S6 and S0 samples. However, the observed upregulation of specific miRNAs in S6 samples likely inhibits these pathways post-transcriptionally, highlighting the role of miRNAs as key regulators of gene expression. These findings underscore the intricate interplay between DNA methylation and miRNA-mediated regulation in sperm development. While hypomethylation enables transcriptional activity, miRNAs add a critical layer of post-transcriptional control, fine-tuning gene expression. This dual mechanism may explain the impaired phenotype observed in S0 samples and emphasizes the importance of miRNAs in maintaining proper sperm function and fertility.

Our findings also underscore the importance of miRNAs as biomarkers of sperm quality. In addition, the underlying mechanisms should be thoroughly investigated. Pathway analysis of S6-miRNA targets revealed their biological significance in the MAPK signaling pathway, mTOR signaling pathway, and cell adhesion molecules. Many studies suggest that the MAPK pathway plays a role in germ cell apoptosis [65]. In mature spermatozoa, this pathway has been linked to capacitation and acrosome reaction [65,66]. The MAPK pathway also contributes to the phosphorylation of heat shock proteins (HSP). Elevated HSP levels in spermatozoa have been correlated with poor blastocyst development [67] and increased sperm DNA fragmentation [68]. Therefore, mTOR signaling and oxidative stress regulation in S6 samples may protect sperm from damage. Previous studies showed that mTOR inhibition triggers autophagy in rat testes, enhancing sperm motility and preventing mitochondrial damage, thus reducing reactive oxygen species production [69,70,71]. The present study is the first to link MAPK and mTOR signaling regulation to S6 sperm. Lastly, S6-miRNAs could be molecular targets for the diagnosis of male infertility and reproductive system disorders, but a deeper understanding of how they regulate reproductive functions is still required.

While miRNA profiling is costlier and more complex than morphology assessment, it provides unique insights into molecular dysfunctions, particularly in subclinical infertility cases with normal morphology. Its ability to uncover regulatory disruptions justifies its use in unexplained cases. However, more research in larger cohorts and with standardized methodologies is required to clarify the associations found in this study. Additionally, animal models are necessary to establish causal relationships for therapeutic development. Ongoing technological advancements, particularly in omics technologies, may facilitate these efforts. Ultimately, the identified miRNAs could serve as biomarkers to enhance IVF success rates and monitor environmental exposures, potentially linking miRNA expression to epigenetic modifications [72].

## 5. Conclusions

This study revealed distinct miRNA expression profiles between S6 and S0 sperm samples from patients undergoing ICSI for male infertility. While further research is required to explore the potential of miRNAs as translational biomarkers for evaluating sperm quality in humans, our findings offer valuable insights into the role of miRNAs in sperm quality.

## Data Availability

The data used to support the findings of this study are included in the article and available from the corresponding author upon request.

## Supplementary Materials

The following supporting information can be downloaded at: https://www.mdpi.com/article/10.3390/genes16010053/s1, Table S1: Upregulated miRNAs in S6 sperm samples compared with S0 samples. Table S2: Downregulated miRNAs in S6 sperm samples compared with S0 samples. Table S3: List of the 910 genes targeted by the 86 miRNAs upregulated in S6 sperm samples, identified by IPA. Table S4: List of 15 genes targeted by the 21 miRNAs downregulated in S6 sperm samples, identified by IPA. Figure S1: Sample collections and sperm parameters.

## References

[1] Eisenberg M.L., Esteves S.C., Lamb D.J., Hotaling J.M., Giwercman A., Hwang K., Cheng Y.-S. (2023). Male Infertility. Nat. Rev. Dis. Primers.

[2] Barratt C.L.R., Björndahl L., De Jonge C.J., Lamb D.J., Osorio Martini F., McLachlan R., Oates R.D., van der Poel S., St John B., Sigman M. (2017). The Diagnosis of Male Infertility: An Analysis of the Evidence to Support the Development of Global WHO Guidance-Challenges and Future Research Opportunities. Hum. Reprod. Update.

[3] Cassuto N.G., Piquemal D., Boitrelle F., Larue L., Lédée N., Hatem G., Ruoso L., Bouret D., Siffroi J.-P., Rouen A. (2021). Molecular Profiling of Spermatozoa Reveals Correlations between Morphology and Gene Expression: A Novel Biomarker Panel for Male Infertility. BioMed Res. Int..

[4] Bansal S.K., Gupta N., Sankhwar S.N., Rajender S. (2015). Differential Genes Expression between Fertile and Infertile Spermatozoa Revealed by Transcriptome Analysis. PLoS ONE.

[5] Martínez-Heredia J., de Mateo S., Vidal-Taboada J.M., Ballescà J.L., Oliva R. (2008). Identification of Proteomic Differences in Asthenozoospermic Sperm Samples. Hum. Reprod..

[6] Moscatelli N., Lunetti P., Braccia C., Armirotti A., Pisanello F., De Vittorio M., Zara V., Ferramosca A. (2019). Comparative Proteomic Analysis of Proteins Involved in Bioenergetics Pathways Associated with Human Sperm Motility. Int. J. Mol. Sci..

[7] Walker W.H. (2022). Regulation of Mammalian Spermatogenesis by miRNAs. Semin. Cell Dev. Biol..

[8] Chen X., Li X., Guo J., Zhang P., Zeng W. (2017). The Roles of microRNAs in Regulation of Mammalian Spermatogenesis. J. Anim. Sci. Biotechnol..

[9] Cassuto N.G., Bouret D., Plouchart J.M., Jellad S., Vanderzwalmen P., Balet R., Larue L., Barak Y. (2009). A New Real-Time Morphology Classification for Human Spermatozoa: A Link for Fertilization and Improved Embryo Quality. Fertil. Steril..

[10] Cassuto N.G., Hazout A., Hammoud I., Balet R., Bouret D., Barak Y., Jellad S., Plouchart J.M., Selva J., Yazbeck C. (2012). Correlation between DNA Defect and Sperm-Head Morphology. Reprod. Biomed. Online.

[11] Cassuto N.G., Montjean D., Siffroi J.-P., Bouret D., Marzouk F., Copin H., Benkhalifa M. (2016). Different Levels of DNA Methylation Detected in Human Sperms after Morphological Selection Using High Magnification Microscopy. BioMed Res. Int..

[12] Ostermeier G.C., Goodrich R.J., Moldenhauer J.S., Diamond M.P., Krawetz S.A. (2005). A Suite of Novel Human Spermatozoal RNAs. J. Androl..

[13] Krawetz S.A., Kruger A., Lalancette C., Tagett R., Anton E., Draghici S., Diamond M.P. (2011). A Survey of Small RNAs in Human Sperm. Hum. Reprod..

[14] Schuster A., Tang C., Xie Y., Ortogero N., Yuan S., Yan W. (2016). SpermBase: A Database for Sperm-Borne RNA Contents. Biol. Reprod..

[15] Ha M., Kim V.N. (2014). Regulation of microRNA Biogenesis. Nat. Rev. Mol. Cell Biol..

[16] Bartel D.P. (2009). MicroRNAs: Target Recognition and Regulatory Functions. Cell.

[17] Bouhallier F., Allioli N., Lavial F., Chalmel F., Perrard M.-H., Durand P., Samarut J., Pain B., Rouault J.-P. (2010). Role of miR-34c microRNA in the Late Steps of Spermatogenesis. RNA.

[18] Belleannée C., Calvo E., Thimon V., Cyr D.G., Légaré C., Garneau L., Sullivan R. (2012). Role of microRNAs in Controlling Gene Expression in Different Segments of the Human Epididymis. PLoS ONE.

[19] Muñoz X., Mata A., Bassas L., Larriba S. (2015). Altered miRNA Signature of Developing Germ-Cells in Infertile Patients Relates to the Severity of Spermatogenic Failure and Persists in Spermatozoa. Sci. Rep..

[20] Salas-Huetos A., James E.R., Aston K.I., Carrell D.T., Jenkins T.G., Yeste M. (2020). The Role of miRNAs in Male Human Reproduction: A Systematic Review. Andrology.

[21] Barad O., Meiri E., Avniel A., Aharonov R., Barzilai A., Bentwich I., Einav U., Gilad S., Hurban P., Karov Y. (2004). MicroRNA Expression Detected by Oligonucleotide Microarrays: System Establishment and Expression Profiling in Human Tissues. Genome Res..

[22] Yang Q., Hua J., Wang L., Xu B., Zhang H., Ye N., Zhang Z., Yu D., Cooke H.J., Zhang Y. (2013). MicroRNA and piRNA Profiles in Normal Human Testis Detected by next Generation Sequencing. PLoS ONE.

[23] Pantano L., Jodar M., Bak M., Ballescà J.L., Tommerup N., Oliva R., Vavouri T. (2015). The Small RNA Content of Human Sperm Reveals Pseudogene-Derived piRNAs Complementary to Protein-Coding Genes. RNA.

[24] Abu-Halima M., Hammadeh M., Schmitt J., Leidinger P., Keller A., Meese E., Backes C. (2013). Altered microRNA Expression Profiles of Human Spermatozoa in Patients with Different Spermatogenic Impairments. Fertil. Steril..

[25] Liu T., Cheng W., Gao Y., Wang H., Liu Z. (2012). Microarray Analysis of microRNA Expression Patterns in the Semen of Infertile Men with Semen Abnormalities. Mol. Med. Rep..

[26] Sánchez-Álvarez J., Cano-Corres R., Fuentes-Arderiu X. (2012). A Complement for the WHO Laboratory Manual for the Examination and Processing of Human Semen (First Edition, 2010). EJIFCC.

[27] Liu C.-G., Calin G.A., Volinia S., Croce C.M. (2008). MicroRNA Expression Profiling Using Microarrays. Nat. Protoc..

[28] Ramírez F., Ryan D.P., Grüning B., Bhardwaj V., Kilpert F., Richter A.S., Heyne S., Dündar F., Manke T. (2016). deepTools2: A next Generation Web Server for Deep-Sequencing Data Analysis. Nucleic Acids Res..

[29] Nelson J.W., Sklenar J., Barnes A.P., Minnier J. (2017). The START App: A Web-Based RNAseq Analysis and Visualization Resource. Bioinformatics.

[30] GTEx Consortium, Laboratory, Data Analysis & Coordinating Center (LDACC)—Analysis Working Group, Statistical Methods Groups—Analysis Working Group, Enhancing GTEx (eGTEx) Groups, NIH Common Fund, NIH/NCI, NIH/NHGRI, NIH/NIMH, NIH/NIDA, Biospecimen Collection Source Site—NDRI (2017). Genetic Effects on Gene Expression across Human Tissues. Nature.

[31] GTEx Consortium Human Genomics (2015). The Genotype-Tissue Expression (GTEx) Pilot Analysis: Multitissue Gene Regulation in Humans. Science.

[32] Hu Z., Shen W.-J., Kraemer F.B., Azhar S. (2017). Regulation of Adrenal and Ovarian Steroidogenesis by miR-132. J. Mol. Endocrinol..

[33] Wang L., Chen G., Wu S., Xu Y., Guo C., Wang M., Liang T., Guo Z., Di H.-J., Hu Z. (2022). Genipin Improves Lipid Metabolism and Sperm Parametersin Obese Mice via Regulation of miR-132 Expression. Acta Biochim. Biophys. Sin. (Shanghai).

[34] Hua M., Liu W., Chen Y., Zhang F., Xu B., Liu S., Chen G., Shi H., Wu L. (2019). Identification of Small Non-Coding RNAs as Sperm Quality Biomarkers for in Vitro Fertilization. Cell Discov..

[35] Xu H., Wang X., Wang Z., Li J., Xu Z., Miao M., Chen G., Lei X., Wu J., Shi H. (2020). MicroRNA Expression Profile Analysis in Sperm Reveals Hsa-Mir-191 as an Auspicious Omen of in Vitro Fertilization. BMC Genom..

[36] Eikmans M., D. H. Anholts J., Blijleven L., Meuleman T., van Beelen E., van der Hoorn M.-L.P., Claas F.H.J. (2020). Optimization of microRNA Acquirement from Seminal Plasma and Identification of Diminished Seminal microRNA-34b as Indicator of Low Semen Concentration. Int. J. Mol. Sci..

[37] Comazzetto S., Di Giacomo M., Rasmussen K.D., Much C., Azzi C., Perlas E., Morgan M., O’Carroll D. (2014). Oligoasthenoteratozoospermia and Infertility in Mice Deficient for miR-34b/c and miR-449 Loci. PLoS Genet..

[38] Yu Z., Raabe T., Hecht N.B. (2005). MicroRNA Mirn122a Reduces Expression of the Posttranscriptionally Regulated Germ Cell Transition Protein 2 (Tnp2) Messenger RNA (mRNA) by mRNA Cleavage. Biol. Reprod..

[39] Boitrelle F., Ferfouri F., Petit J.M., Segretain D., Tourain C., Bergere M., Bailly M., Vialard F., Albert M., Selva J. (2011). Large Human Sperm Vacuoles Observed in Motile Spermatozoa under High Magnification: Nuclear Thumbprints Linked to Failure of Chromatin Condensation. Hum. Reprod..

[40] Abu-Halima M., Hammadeh M., Backes C., Fischer U., Leidinger P., Lubbad A.M., Keller A., Meese E. (2014). Panel of Five microRNAs as Potential Biomarkers for the Diagnosis and Assessment of Male Infertility. Fertil. Steril..

[41] Liu W.-M., Pang R.T.K., Chiu P.C.N., Wong B.P.C., Lao K., Lee K.-F., Yeung W.S.B. (2012). Sperm-Borne microRNA-34c Is Required for the First Cleavage Division in Mouse. Proc. Natl. Acad. Sci. USA.

[42] Cui L., Fang L., Shi B., Qiu S., Ye Y. (2015). Spermatozoa Micro Ribonucleic Acid-34c Level Is Correlated with Intracytoplasmic Sperm Injection Outcomes. Fertil. Steril..

[43] Wang C., Yang C., Chen X., Yao B., Yang C., Zhu C., Li L., Wang J., Li X., Shao Y. (2011). Altered Profile of Seminal Plasma microRNAs in the Molecular Diagnosis of Male Infertility. Clin. Chem..

[44] Salas-Huetos A., Blanco J., Vidal F., Godo A., Grossmann M., Pons M.C., F-Fernández S., Garrido N., Anton E. (2015). Spermatozoa from Patients with Seminal Alterations Exhibit a Differential Micro-Ribonucleic Acid Profile. Fertil. Steril..

[45] Salas-Huetos A., Blanco J., Vidal F., Grossmann M., Pons M.C., Garrido N., Anton E. (2016). Spermatozoa from Normozoospermic Fertile and Infertile Individuals Convey a Distinct miRNA Cargo. Andrology.

[46] Gholami D., Amirmahani F., Yazdi R.S., Hasheminia T., Teimori H. (2021). MiR-182-5p, MiR-192-5p, and MiR-493-5p Constitute a Regulatory Network with CRISP3 in Seminal Plasma Fluid of Teratozoospermia Patients. Reprod. Sci..

[47] Reza A.M.M.T., Choi Y.-J., Han S.G., Song H., Park C., Hong K., Kim J.-H. (2019). Roles of microRNAs in Mammalian Reproduction: From the Commitment of Germ Cells to Peri-Implantation Embryos. Biol. Rev. Camb. Philos. Soc..

[48] Jung Y.H., Gupta M.K., Shin J.Y., Uhm S.J., Lee H.T. (2010). MicroRNA Signature in Testes-Derived Male Germ-Line Stem Cells. Mol. Hum. Reprod..

[49] Wu W., Hu Z., Qin Y., Dong J., Dai J., Lu C., Zhang W., Shen H., Xia Y., Wang X. (2012). Seminal Plasma microRNAs: Potential Biomarkers for Spermatogenesis Status. Mol. Hum. Reprod..

[50] Martins R.P., Krawetz S.A. (2005). RNA in Human Sperm. Asian J. Androl..

[51] Yamamoto C.M., Hikim A.P., Lue Y., Portugal A.M., Guo T.B., Hsu S.Y., Salameh W.A., Wang C., Hsueh A.J., Swerdloff R.S. (2001). Impairment of Spermatogenesis in Transgenic Mice with Selective Overexpression of Bcl-2 in the Somatic Cells of the Testis. J. Androl..

[52] Yan N., Lu Y., Sun H., Qiu W., Tao D., Liu Y., Chen H., Yang Y., Zhang S., Li X. (2009). Microarray Profiling of microRNAs Expressed in Testis Tissues of Developing Primates. J. Assist. Reprod. Genet..

[53] Cimmino A., Calin G.A., Fabbri M., Iorio M.V., Ferracin M., Shimizu M., Wojcik S.E., Aqeilan R.I., Zupo S., Dono M. (2005). miR-15 and miR-16 Induce Apoptosis by Targeting BCL2. Proc. Natl. Acad. Sci. USA.

[54] Grunewald S., Sharma R., Paasch U., Glander H.-J., Agarwal A. (2009). Impact of Caspase Activation in Human Spermatozoa. Microsc. Res. Tech..

[55] Paasch U., Grunewald S., Dathe S., Glander H.-J. (2004). Mitochondria of Human Spermatozoa Are Preferentially Susceptible to Apoptosis. Ann. N. Y. Acad. Sci..

[56] Sakkas D., Ramalingam M., Garrido N., Barratt C.L.R. (2015). Sperm Selection in Natural Conception: What Can We Learn from Mother Nature to Improve Assisted Reproduction Outcomes?. Hum. Reprod. Update.

[57] Tian H., Lv M., Li Z., Peng D., Tan Y., Wang H., Li Q., Li F., Liang W. (2018). Semen-Specific miRNAs: Suitable for the Distinction of Infertile Semen in the Body Fluid Identification?. Forensic Sci. Int. Genet..

[58] Mostafa T., Rashed L.A., Nabil N.I., Osman I., Mostafa R., Farag M. (2016). Seminal miRNA Relationship with Apoptotic Markers and Oxidative Stress in Infertile Men with Varicocele. BioMed Res. Int..

[59] Curry E., Safranski T.J., Pratt S.L. (2011). Differential Expression of Porcine Sperm microRNAs and Their Association with Sperm Morphology and Motility. Theriogenology.

[60] Rainey M.A., George M., Ying G., Akakura R., Burgess D.J., Siefker E., Bargar T., Doglio L., Crawford S.E., Todd G.L. (2010). The Endocytic Recycling Regulator EHD1 Is Essential for Spermatogenesis and Male Fertility in Mice. BMC Dev. Biol..

[61] Mendis S.H.S., Meachem S.J., Sarraj M.A., Loveland K.L. (2011). Activin A Balances Sertoli and Germ Cell Proliferation in the Fetal Mouse Testis. Biol. Reprod..

[62] Li L., Zhu Y., Chen T., Sun J., Luo J., Shu G., Wang S., Zhu X., Jiang Q., Zhang Y. (2019). MiR-125b-2 Knockout in Testis Is Associated with Targeting to the PAP Gene, Mitochondrial Copy Number, and Impaired Sperm Quality. Int. J. Mol. Sci..

[63] Liu B., Cui Y., Chen W., Du L., Li C., Wan C., He Z. (2021). Hsa-miR-100-3p Controls the Proliferation, DNA Synthesis, and Apoptosis of Human Sertoli Cells by Binding to SGK3. Front. Cell Dev. Biol..

[64] Gupta A., Vats A., Ghosal A., Mandal K., Sarkar R., Bhattacharya I., Das S., Pal R., Majumdar S.S. (2022). Follicle-Stimulating Hormone-Mediated Decline in miR-92a-3p Expression in Pubertal Mice Sertoli Cells Is Crucial for Germ Cell Differentiation and Fertility. Cell. Mol. Life Sci..

[65] Almog T., Naor Z. (2010). The Role of Mitogen Activated Protein Kinase (MAPK) in Sperm Functions. Mol. Cell. Endocrinol..

[66] Almog T., Lazar S., Reiss N., Etkovitz N., Milch E., Rahamim N., Dobkin-Bekman M., Rotem R., Kalina M., Ramon J. (2008). Identification of Extracellular Signal-Regulated Kinase 1/2 and P38 MAPK as Regulators of Human Sperm Motility and Acrosome Reaction and as Predictors of Poor Spermatozoan Quality. J. Biol. Chem..

[67] McReynolds S., Dzieciatkowska M., Stevens J., Hansen K.C., Schoolcraft W.B., Katz-Jaffe M.G. (2014). Toward the Identification of a Subset of Unexplained Infertility: A Sperm Proteomic Approach. Fertil. Steril..

[68] Intasqui P., Camargo M., Del Giudice P.T., Spaine D.M., Carvalho V.M., Cardozo K.H.M., Cedenho A.P., Bertolla R.P. (2013). Unraveling the Sperm Proteome and Post-Genomic Pathways Associated with Sperm Nuclear DNA Fragmentation. J. Assist. Reprod. Genet..

[69] Liu S., Huang L., Geng Y., He J., Chen X., Xu H., Li R., Wang Y., Ding Y., Liu X. (2017). Rapamycin Inhibits Spermatogenesis by Changing the Autophagy Status through Suppressing Mechanistic Target of Rapamycin-p70S6 Kinase in Male Rats. Mol. Med. Rep..

[70] Aparicio I.M., Espino J., Bejarano I., Gallardo-Soler A., Campo M.L., Salido G.M., Pariente J.A., Peña F.J., Tapia J.A. (2016). Autophagy-Related Proteins Are Functionally Active in Human Spermatozoa and May Be Involved in the Regulation of Cell Survival and Motility. Sci. Rep..

[71] Silva J.V., Cabral M., Correia B.R., Carvalho P., Sousa M., Oliveira P.F., Fardilha M. (2019). mTOR Signaling Pathway Regulates Sperm Quality in Older Men. Cells.

[72] Bendayan M., Caceres L., Saïs E., Swierkowski-Blanchard N., Alter L., Bonnet-Garnier A., Boitrelle F. (2022). Human Sperm Morphology as a Marker of Its Nuclear Quality and Epigenetic Pattern. Cells.

