# Supplementary figures and images for: Genome-Wide microRNA Expression Profiling in Human Spermatozoa and Its Relation to Sperm Quality
Source: Genes (Basel). 2025 Jan 4;16(1):53. doi: 10.3390/genes16010053 (PMC11765444; doi:10.3390/genes16010053)

## Slide 1
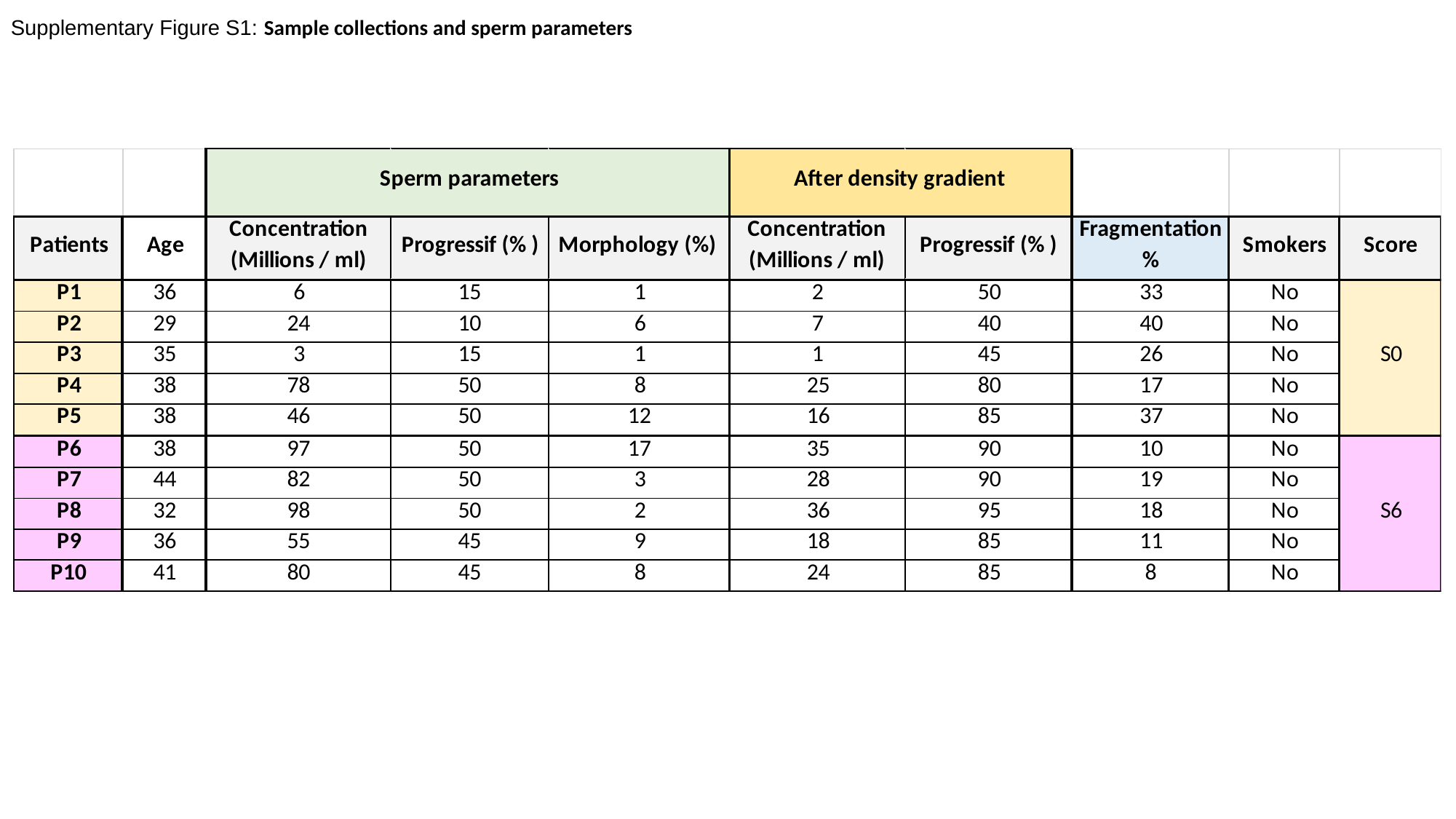

Supplementary Figure S1: Sample collections and sperm parameters

Supplement: Supplementary file 1 [file genes-16-00053-s001.zip › genes-3361727-Figure S1.pptx]
